# Correlation of MLASA2 Clinical Phenotype and Survival with Mt-TyrRS Protein Damage: Linking Systematic Review, Meta-Analysis and 3D Hotspot Mapping

**DOI:** 10.3390/cimb48010095

**Published:** 2026-01-16

**Authors:** José Rafael Villafan-Bernal, Angélica Martínez-Hernández, Humberto García-Ortiz, Cecilia Contreras-Cubas, Israel Guerrero-Contreras, José Luis Frías-Cabrera, Federico Centeno-Cruz, Monserrat Ivonne Morales Rivera, Jhonatan Rosas Hernández, Alessandra Carnevale, Francisco Barajas-Olmos, Lorena Orozco

**Affiliations:** 1 Immunogenomics and Metabolic Diseases Laboratory, Instituto Nacional de Medicina Genómica, Secretaría de Salud, Mexico City 14610, Mexico; joravibe@hotmail.com (J.R.V.-B.); amartinez@inmegen.gob.mx (A.M.-H.); hgarcia@inmegen.gob.mx (H.G.-O.); ccontreras@inmegen.gob.mx (C.C.-C.); israel.guerrerocon@gmail.com (I.G.-C.); joseluisfrica@gmail.com (J.L.F.-C.); fcenteno@inmegen.gob.mx (F.C.-C.);; 2 Investigador por México, Secretaría de Humanidades Ciencia, Tecnología e Innovación, Mexico City 03940, Mexico; 3 Centro de Rehabilitación Infantil, Teletón, Altamira 89607, Mexico; dr.rosas.genetica@outlook.com; 4 Mendelian Diseases Laboratory, Instituto Nacional de Medicina Genómica, Secretaría de Salud, Mexico City 14610, Mexico; acarnevale@inmegen.gob.mx

**Keywords:** MLASA2, survival, *YARS2*, meta-analysis, phenotypes, 3D hotspot mapping, mitochondrial disorder

## Abstract

Myopathy, Lactic Acidosis, and Sideroblastic Anemia type 2 (MLASA2) is a rare mitochondrial disorder caused by pathogenic variants (PVs) in the *YARS2* gene (which encodes the Mt-TyrRS protein. We performed a comprehensive clinical–molecular synthesis by integrating a systematic review and meta-analysis of all published MLASA2 cases with survival modeling and three-dimensional structural mapping. Across the aggregated cohort, anemia (88.6%), sideroblastic phenotype (85.7%), and lactic acidosis (82.9%) were the most prevalent phenotypes. Fifteen PVs were identified, dominated by p.(Phe52Leu) (29.4%). Survival estimates were 94.1% at 10 years, 70.7% at 30 years, and 42.4% at 50 years; cardiomyopathy and diagnosis before age 10 were associated with decreased survival. We generated the first 3D structural map of all reported Mt-TyrRS PVs, identifying nine spatial hotspots across catalytic, anticodon-binding, and tRNA-binding domains. An integrated framework combining structural density, clinical severity, in silico predictions, and ΔΔG destabilization classified three clusters as High-risk, three as Medium-risk, and three as Low-risk. Among them, cluster 3, a large catalytic hotspot encompassing 44 residues and including nearly half of all MLASA2 cases, showed the strongest pathogenic convergence. This clinical–structural integration provides new insights for a better comprehension of MLASA2, enhancing variant interpretation and improving diagnostic and prognostic precision.

## 1. Introduction

Myopathy, Lactic Acidosis, and Sideroblastic Anemia (MLASA) syndrome is a rare mitochondrial-related disorder with an estimated prevalence of <1 per 1,000,000 [1] and marked phenotypic heterogeneity, with typical onset during the first decade of life [2]. Core clinical features include myopathy, lactic acidosis, sideroblastic anemia, and exercise intolerance, though associated manifestations may include facial dysmorphism and cardiomyopathy [3]. Three genetic subtypes of MLASA syndrome have been identified: MLASA Type 1, caused by PVs in the Pseudouridine Synthase 1 gene (*PUS1*) [4]; MLASA Type 2 (MLASA2), caused by PVs in the tyrosyl-tRNA synthetase 2 gene (*YARS2*) [5]; and MLASA Type 3, resulting from PVs in the mitochondrial *ATP6* gene [6].

MLASA2 distinguishes itself from other mitochondrial aminoacyl-tRNA synthetase disorders, as it predominantly manifests with musculoskeletal abnormalities rather than central nervous system (CNS) involvement [7]. The *YARS2* gene encodes the mitochondrial tyrosyl-tRNA synthetase (Mt-TyrRS), a protein essential for the binding of tyrosine to its analogous tRNA within the mitochondria [8]. Although Mt-TyrRS is ubiquitously expressed across tissues, including heart, lung, bone marrow, skeletal muscle, and the central nervous system [9,10], the selective vulnerability observed in MLASA2 reflects tissue-specific bioenergetic thresholds during development rather than restricted gene expression [11,12]. Consistent with this, Riley et al. reported reduced *YARS2* protein levels and respiratory chain subunits in skeletal muscle from affected individuals, whereas patient-derived fibroblasts showed preserved protein levels and function [7]. High-energy tissues such as skeletal muscle and hematopoietic lineages, characterized by high mitochondrial density and continuous mitochondrial protein synthesis, are therefore particularly susceptible to partial defects in mitochondrial translation [13,14]. In contrast, neurons may retain greater compensatory capacity [15], potentially explaining the relative sparing of neurological involvement in most MLASA2 cases.

Despite growing recognition of MLASA2, the accurate frequency of its clinical phenotypes remains uncertain, owing to its rarity, variable expressivity, and the scattered nature of published case reports. Likewise, no systematic evaluation of survival has been performed, and the three-dimensional distribution of *YARS2* PVs—a key element for understanding molecular mechanisms—has never been mapped.

To address these gaps, we conducted a comprehensive synthesis integrating: a systematic review and meta-analysis of MLASA2 phenotypes; survival analysis of all reported cases; three-dimensional structural hotspot mapping of *YARS2*; and the correlation of 3D structural hotspots with clinical manifestations and survival. With this integrated approach, we expect to provide new insights into genotype–phenotype relationships, providing a framework for the interpretability of new or uncertain significance (VUS) *YARS2* PVs and their prognosis, which remain as a bottleneck in the diagnosis of mitochondrial diseases.

## 2. Materials and Methods

### 2.1. Systematic Review and Meta-Analysis

This systematic review and meta-analysis were performed to estimate the pooled prevalence of MLASA2 clinical manifestations, following Cochrane and PRISMA (Preferred Reporting Items for Systematic Reviews and Meta-Analyses) recommendations [16]. No protocol registration was performed because PROSPERO was unavailable for registering protocols for meta-analyses other than clinical trials.

The systematic literature search was carried out in PubMed (https://pubmed.ncbi.nlm.nih.gov/, accessed on 9 January 2026), Scopus (https://www.elsevier.com/es-es/products/scopus, accessed on 9 January 2026), and Web of Science (https://access.clarivate.com/, accessed on 9 January 2026) databases in September 2024. The Query [Appendix A.1] was re-run in January 2026. Two reviewers (I.G.C. and H.G.-O.) independently screened the titles and abstracts retrieved from databases to assess their eligibility according to predefined inclusion and exclusion criteria. The inclusion criteria were that the article reported at least one case of MLASA2, with a preference for reporting the PV responsible for the disease. Studies performed in species other than humans or focused on a different type of MLASA were excluded. Full texts of potentially relevant articles were then evaluated in detail by the same reviewers. Any discrepancies were resolved by consensus, and a third reviewer (A.M.-H. or C.C.-C.) was consulted when disagreement persisted. No automation tools were used in the selection process.

Data extraction was performed independently by two reviewers (I.G.C. and J.R.V.-B.) using a standardized data collection form designed for this review. Extracted information included study characteristics, clinical manifestations, PVs, and survival data.

The pooled prevalence of each clinical manifestation was estimated using a random-effects model (REM) weighting by the inverse of the variance of logit-transformed proportions, implemented through the metaprop function of the meta package (version 8.1) in R. Pooled prevalence estimates with 95% confidence intervals (CIs) were then back-transformed from the logit scale to the original proportion scale for interpretation. The synthesis of the pooled prevalence for each clinical manifestation is presented in a figure, along with its 95% confidence intervals.

Between-study variance (τ^2^) was estimated by maximum likelihood, and heterogeneity was summarized with τ^2^, τ, H, and reported as I^2^. Global heterogeneity was assessed via Q-based tests (Wald and likelihood-ratio), and pooled estimates with 95% CIs were back transformed to the proportion scale.

To evaluate the robustness of the pooled prevalence estimates, we conducted sensitivity analyses. First, leave-one-out analyses were performed by sequentially removing each study and recalculating the pooled prevalence to assess the influence of each study. Second, we compared alternative continuity corrections for zero-event cells, contrasting the primary approach (adding 0.5 to all studies) with a study-specific correction applied only when a study contained zero or all events. Finally, we examined the impact of different transformations by comparing the primary logit transformation (PLOGIT) with the Freeman–Tukey double arcsine transformation (PFT).

The quality of case and case series reports was evaluated using the Joanna Briggs Institute (JBI) tools [17]. The results of the meta-analysis are presented in tables or figures as appropriate.

### 2.2. Survival Analysis

For survival analysis, the retrieved information included event [dead or survival], time [age at the last follow-up], and variables for the Log-Rank test to identify factors influencing survival, including sex, age at diagnosis, transfusion dependency, myopathy, lactic acidosis, cardiomyopathy, respiratory insufficiency, pathogenic variant, zygosity, and affected protein domain. Differences in data extraction were resolved by consensus, and when necessary, by consulting a third reviewer (A.M.-H. or C.C.-C.). No automated data extraction tools were applied, and study investigators were not contacted for additional information. In case of missing data, it was reported. In the survival analysis, from the 36 cases reported in the literature, we included 34 patients with confirmed PV in *YARS2* since two siblings of index cases had no identified PV, only clinical features compatible with MLASA2 [one from Rawles’s and another from Sommerville’s studies].

### 2.3. Structural Evaluation of YARS2 Hotspot Clusters and Hotspot Prioritization

#### 2.3.1. Variant Identification and Annotation

All *YARS2* PVs included in this study were compiled from published case reports and the gnomAD database. Variant nomenclature was standardized according to HGVS recommendations. Functional impact annotations included modern machine-learning pathogenicity predictors: REVEL, AlphaMissense, ESM1b, while structural destabilization was inferred from predicted ΔΔG values generated by Dynamut2. PVs were mapped to the canonical Mt-TyrRS domains, including the catalytic core, anticodon-binding region, and tRNA-binding (tRNAb) domain.

#### 2.3.2. Structural-Cluster Identification

Structural hotspot detection in Mt-TyrRS was performed through a multistep procedure integrating curated variant mapping, extraction of three-dimensional coordinates from AlphaFold, and graph-based spatial clustering.

All residues corresponding to reported PVs in the systematic literature review (missense and non-missense) and gnomAD variants (pathogenic, likely pathogenic, and of conflicting results of pathogenicity) were first compiled and mapped onto the monomeric AlphaFold model AF-Q9Y2Z4-F1 using PyMOL (version 2.7). Residue positions were selected and reduced to their Cα atoms to provide a standardized structural representation. This set of Cα coordinates served as the input for spatial analysis [18].

Pairwise Euclidean distances were computed for all residue pairs. A spatial adjacency matrix was then constructed by defining “structural proximity” as a Cα–Cα distance <12 Å, a threshold consistent with established criteria for detecting local structural neighborhoods within globular proteins [15]. Residue pairs falling below this threshold were considered directly connected in three-dimensional space.

Clusters were identified by performing a connected-component search on the adjacency matrix. This approach groups residues into structurally coherent sets in which every residue can be reached from any other through a chain of <12 Å contacts [19]. Each connected component was defined as a candidate hotspot.

Cluster compactness was quantified by recalculating all intracluster Cα–Cα distances and averaging only those below the cut-off. These mean distances were used to characterize the internal geometry of each hotspot. A complete cluster summary, including size, residue composition, and mean intra-cluster distances, was exported for downstream functional and clinical correlation analyses. Identified clusters were visualized in ChimeraX, assigned unique colors, and rendered using stick representation.

This workflow enabled objective delineation of spatially defined mutation hotspots within Mt-TyrRS and provided a reproducible framework for integrating structural, functional in silico, and clinical data.

#### 2.3.3. Hotspot and Cluster Characterization

To determine the biological and clinical significance of each cluster, we applied an integrated multi-axis scoring approach incorporating structural, in silico functional, the number of cases previously reported, and clinical criteria:

C1—Structural cluster: ≥3 residues [20]
C2—REVEL enrichment: ≥50% of PVs with REVEL ≥0.5 [21]
C3—AlphaMissense enrichment: ≥50% variants classified as ‘P’ (pathogenic) [22]
C4—ESM1b enrichment: ≥50% variants classified as ‘D’ (deleterious) [23]
C5—Structural destabilization: ≥1 variant with |ΔΔG| ≥ 0.5 [24]
C6—Number of cases reported in the literature and CSS: number of reported patients carrying PVs in cluster residues and the Clinical Severity Score (CSS).

The Clinical Severity Score (CSS) was computed per cluster as the mean frequency of major manifestations: myopathy, cardiomyopathy, lactic acidosis, and anemia with transfusion dependence. The CSS was defined as the mean percentage of these manifestations present across patients within a cluster, thereby producing a continuous severity metric ranging from 0 to 100%. For example, if a cluster exhibited myopathy in 80% of patients, cardiomyopathy in 40%, lactic acidosis in 60%, transfusion dependence in 50%, and mortality in 30%, the CSS would be calculated as the average of these five values (in this case, 52%). This scoring system weights all major phenotypes equally and enables a standardized comparison of clinical severity across clusters.

Clusters were categorized in two dimensions to distinguish clinical severity from the strength of pathogenic evidence. Severity of the clinical phenotype was assessed by integrating the Clinical Severity Score (CSS) with the distribution of major MLASA2 manifestations within each cluster, including myopathy, cardiomyopathy, lactic acidosis, and transfusion dependence, as well as reported mortality. Clusters were considered to exhibit high severity when they showed a CSS of ≥0.60 or when severe phenotypes were present in at least half of affected individuals, or when mortality exceeded 30%. Clusters were classified as medium severity when the CSS ranged between 0.30 and 0.59, or when at least one major phenotype was present in ≥30% of cases, or when mortality ranged between 10% and 29%. Clusters with lower CSS values, infrequent major manifestations, and mortality <10% were considered low severity.

Strength of evidence for pathogenicity was evaluated independently from clinical severity and reflected the robustness and reproducibility of data supporting each region. Evidence was considered high when a cluster included five or more reported patients, or when PVs in the region recurred across independent publications, and when at least two functional or structural predictors (REVEL, AlphaMissense, ESM1b, or ΔΔG destabilization) suggested damaging effects. Also, clusters exhibiting ≥80% penetrance of severe multisystem phenotypes together with mortality ≥30% were considered *high-evidence for pathogenic regions*, regardless of sample size, under the rationale that extreme clinical severity reflects strong pathogenic potential even in ultrarare disorders.

Medium evidence for pathogenicity was assigned to clusters containing three to four reported patients or those supported by a single functional predictor together with structural compactness or mortality <20%. Low evidence was assigned to clusters with only one or two reported patients, minimal or absent in silico pathogenic signal, or clusters defined primarily by structural proximity without recurrent clinical cases or mortality.

## 3. Results

### 3.1. Selection and General Characteristics of the Studies

**Figure 1:**
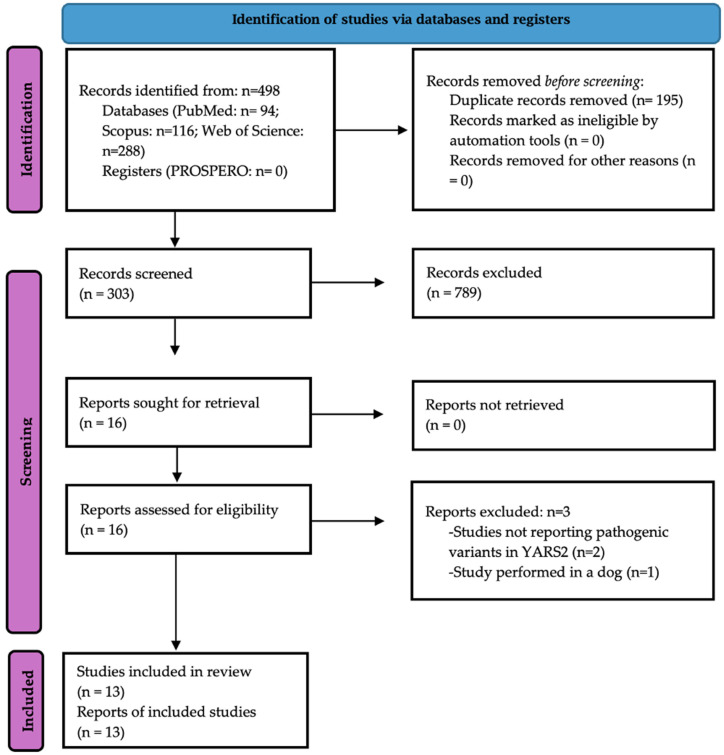
The PRISMA 2020 flow diagram of this study.

The systematic search retrieved 497 studies from electronic databases, and no additional studies were found manually. In total, 16 studies were eligible for full-text review, and 13 were retained for the meta-analysis [7,25,26,27,28,29,30,31,32,33,34,35,36] (Figure 1). Three studies were excluded because they reported PVs in non-*YARS2* genes [6,37] or were not performed in humans [38].

We found 36 cases of MLASA2 syndrome previously reported in the literature. The geographic origins of cases were the USA (n = 14), the United Kingdom (n = 8), Australia (n = 7), Italy (n = 2), Spain (n = 2), Canada (n = 1), and Türkiye (n = 1). Remarkably, 57.1% of cases were of European ancestry and 31.4% of Lebanese descent. The primary methods for PV identification were Sanger sequencing (four studies) and WES or whole-genome sequencing (WGS) (five studies). Most cases (n = 20) had European ancestry, and 9 cases were of Lebanese descent; the remaining cases had diverse ancestries (Table 1). From the 36 cases, two have not reported *YARS2* PV. The spectrum of the 16 Mt-TyrRS PVs found in the included studies is presented in Table 1; the most common is p.(Phe52Leu), observed in 10 from 34 patients (29.4%).

Age at symptoms onset was 0–5 years in 63.9% of patients (n = 23/36), 6–10 years in 5.6% (n = 2/36), 11–19 years in 16.7% and 20 years or older in 13.9% of participants. Most cases of anemia were diagnosed under 5 years (54.6%), 13.6% during school age, 18.2% during adolescence, and 13.6% during adulthood. Importantly, the diagnosis was delayed more than 10 years in 44.4% of cases with available information.

### 3.2. Bias Assessment of Included Studies

According to the JBI critical appraisal tools [17], the quality of case reports and case series was high. For case reports, all studies scored ≥7/8; all case series scored ≥8/10 [Appendix A.2]. These results indicate that the included studies employed robust methodologies, provided transparent reporting, and posed minimal risk of bias, supporting the reliability and credibility of this systematic review’s findings.

### 3.3. Pooled Prevalence of Clinical Manifestations

**Figure 2:**
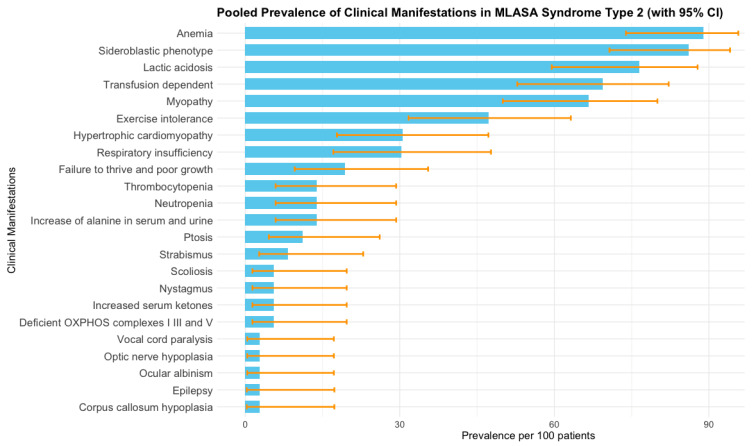
Pooled prevalence of clinical manifestations of MLASA2 obtained by meta-analysis of individual studies [6,7,25,26,27,28,29,30,31,32,33,34,35,36,37,38]. Bars represent prevalence per 100 patients, and error bars correspond to 95% confidence intervals estimated from the meta-analysis. I^2^ was 0% for all clinical manifestations.

The most frequent clinical manifestations identified were anemia [88.9%; (95% CI 73.9–95.7)], with sideroblastic phenotype [86.1% (95% CI 70.7–94.1)] and lactic acidosis [76.5% (95% CI 59.5–87.8)], followed by transfusion-dependency [69.4% (95% CI 52.8–82.2)], myopathy [66.7% (95% CI 50.0–80.0)], and exercise intolerance [47.2% (95% CI 31.7–63.2)]. The pooled prevalences of deficient oxidative phosphorylation (complexes I, III, and V), hypertrophic cardiomyopathy, and failure to thrive were 5.6% (95% CI 1.4–19.7), 30.6% (95% CI 17.8–47.2), and 19.4% (95% CI 9.6–35.5), respectively. Other less common phenotypes were an increase in alanine in serum and urine, hematological abnormalities such as neutropenia and thrombocytopenia, increased serum ketones, and several ocular problems (strabismus, nystagmus, ptosis, ocular albinism, and optic nerve hypoplasia). Vocal cord paralysis, epilepsy, corpus callosum hypoplasia, and scoliosis were each present in only one patient (Figure 2). Across clinical manifestations, pooled prevalence estimates were stable in leave-one-out analyses and insensitive to zero-cell corrections or the choice of transformation (PLOGIT vs. PFT), supporting the robustness of our results even where I^2^ was 0% (Supplementary File S1). No meta-regression was performed because I^2^ was 0%. The sensitivity analysis demonstrated no influence of individual studies on the pooled prevalence of clinical manifestations of MLASA2 (Supplementary File S1).

### 3.4. Survival and Associated Factors

**Figure 3:**
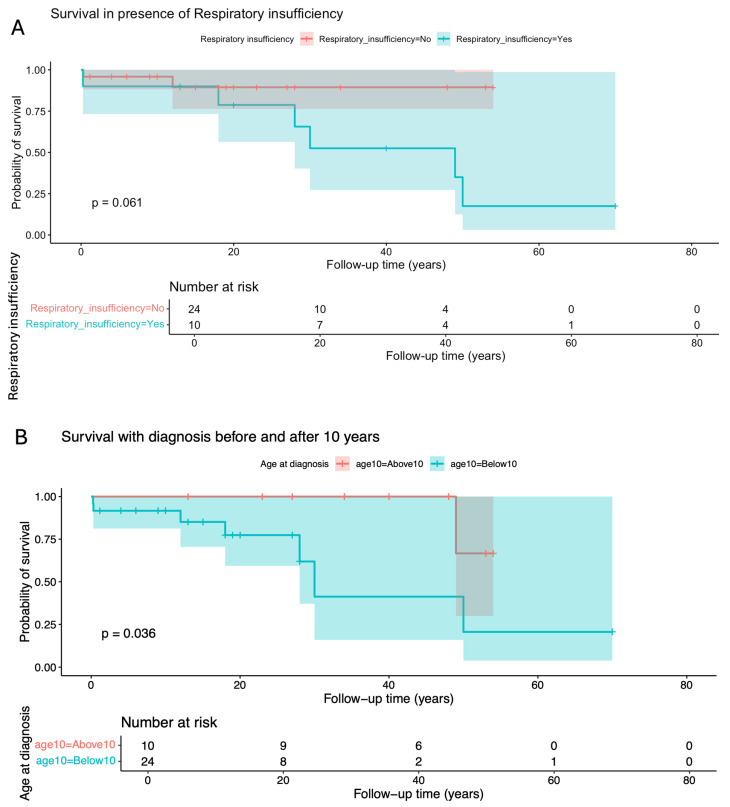
Kaplan–Meier curve and log-rank test for survival probability in the presence or absence of (**A**) respiratory insufficiency and (**B**) according to the age at diagnosis.

**Figure 4:**
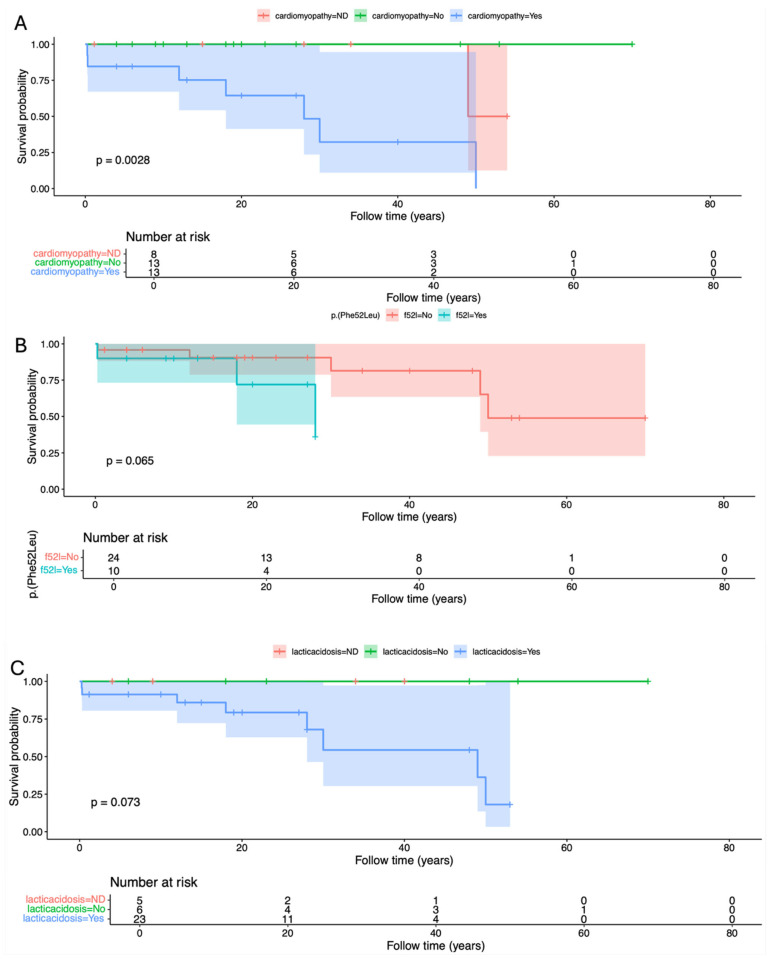
Kaplan–Meier curve and log-rank test for survival probability in the presence and absence of (**A**) cardiomyopathy, (**B**) p.(Phe52Leu), and (**C**) Lactic acidosis.

Cumulative survival proportion was 0.941 (95% IC 0.865–1) at 10 years old, 0.707 (95% IC 0.519–0.964) at 40 years old, and a dramatic reduction to 0.424 (0.195–0.925) at 50 years old (Table 2). Factors significantly or marginally related to lower survival were the presence of cardiomyopathy (*p* = 0.0028), diagnosis before 10 years (*p* = 0.0364), homozygosity for p.(Phe52Leu) (*p* = 0.0655), lactic acidosis (*p* = 0.0734, and respiratory insufficiency (*p* = 0.0614) (Figure 3 and Figure 4).

### 3.5. Structural Evaluation of YARS2 Hotspot Clusters Through 3D Mapping

**Figure 5:**
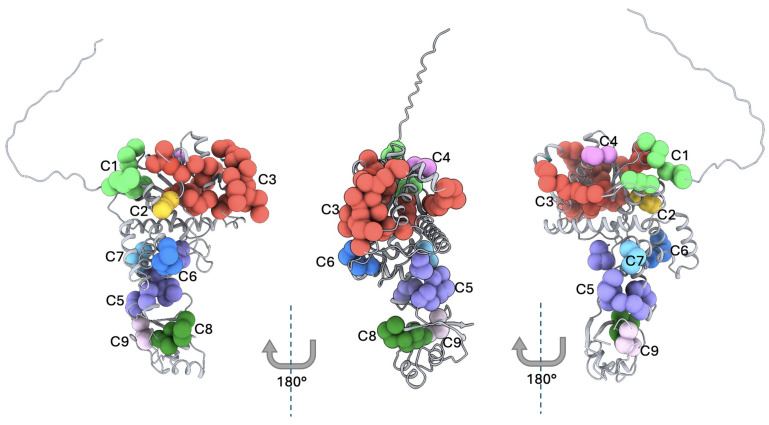
Three-dimensional mapping of the Hotspot Clusters in Mt-TyrRS. Predicted 3D structures for the three representative protein faces of Mt-TyrRS. The figure shows the 9 clusters presented in Mt-TyrRS, where each marker represents a reported pathogenic variant. The monomeric AlphaFold AF-Q9Y2Z4-F1 was used to perform the 3D mapping. C = Cluster from 1 to 9.

Because PVs in *YARS2* tend to affect relatively contiguous amino acid residues within three-dimensional structure rather than being randomly distributed along the linear protein sequence, we pursued a 3D structural mapping approach to better capture their spatial organization and identify hotspots clusters. Such three-dimensional analysis enables the identification of functionally constrained microdomains that may not be apparent from sequence-based inspection alone but are brought into proximity within the folded protein. To achieve a comprehensive and robust assessment, we included all PVs in Mt-TyrRS reported in MLASA2 cases, as well as those classified as pathogenic or likely pathogenic in publicly accessible databases such as gnomAD. This integrative strategy allowed us to define structurally coherent hotspot clusters and to relate spatial aggregation of variants to functional disruption and clinical severity. The three-dimensional mapping of all reported Mt-TyrRS PVs onto its monomeric model AF-Q9Y2Z4-F1 identified nine (C1–C9) spatially cohesive mutation hotspots, each defined by Cα–Cα distances <12 Å. These ranged from compact 3-residue microclusters to a broad 44-residue cluster (Cluster 3). Residues were distributed across the catalytic, anticodon-binding, and tRNA-binding (tRNAb) domains, suggesting that pathogenic variation in *YARS2* is structurally organized rather than randomly scattered [Figure 5 and Table 3].

### 3.6. Hotspot Clusters Prioritization and Its Clinical Correlations

To evaluate pathogenic relevance, each cluster was assessed using two complementary criteria: (1) the prevalence of the main clinical manifestations and (2) strength of pathogenic evidence, defined by recurrence, in silico damage signals, ΔΔG destabilization, and structural compactness. The summary characteristics of each cluster, including protein structure and clinical manifestations, are presented in Table 3.

Cluster 3 emerged as the most extensively supported pathogenic region. Located within the catalytic domain and encompassing 44 residues, it included 17 reported patients—nearly half of all published MLASA2 cases. Clinically, this region showed high severity, with lactic acidosis in 75%, myopathy in 65%, and transfusion dependence in 67% of affected individuals. Strong enrichment in ESM1b and consistent ΔΔG destabilization, together with its dense structural organization, reinforce cluster 3 as the principal catalytic locus underlying MLASA2. Mortality for carriers of PV in this region was 22% [Table 3].

In addition to cluster 3, two further clusters demonstrated high clinical severity combined with high pathogenic evidence. Cluster 2, despite comprising only three residues affected and two reported cases, exhibited maximal clinical severity (CSS = 1.0), with universal myopathy, cardiomyopathy, lactic acidosis, and transfusion dependence, and a mortality rate of 50%. Strong support from REVEL, ESM1b, and ΔΔG analyses indicates that this compact catalytic microdomain is extremely intolerant to variation. Cluster 8, located within the tRNA-binding region, also showed high severity (CSS = 0.917) with universal myopathy, cardiomyopathy, and lactic acidosis, and the highest observed mortality (67%). The three patients affected and consistent functional disruption support its designation as a high-risk microdomain.

Regions with high clinical severity but more moderate supporting evidence included cluster 6, positioned within the anticodon-binding domain. All affected individuals had myopathy and transfusion dependence, and most exhibited lactic acidosis, yielding a CSS of 0.783. Although in silico signals were less extensive and mortality was not observed, the five independent individuals with PV in this region underscores its biological relevance.

Clusters with intermediate pathogenicity included cluster 1, a small catalytic subregion with four affected individuals and moderate clinical severity (CSS = 0.417). PVs in this region produced reproducible but heterogeneous hematologic and muscular involvement. Although functional predictions were modest, the convergence of structural and phenotypic signals supports an intermediate level of pathogenic importance. Cluster 5, spanning residues across the catalytic, anticodon-binding, and tRNA-binding domains, also demonstrated medium clinical severity (CSS = 0.500) and medium evidence of pathogenicity.

Conversely, clusters 4, 7, and 9 showed low clinical severity and low strength of evidence. Cluster 4 exhibited minimal disease involvement despite its catalytic location, and clusters 7 and 9 had no reported cases. These regions may represent structurally cohesive but functionally tolerant motifs within Mt-TyrRS.

Detailed scores for Alpha Missense, REVEL, ESM1b, and 40 other computational predictors are in Supplementary File S1.

Taken together, this two-dimensional integration of structural mapping and clinical data reveals that the pathogenic landscape of *YARS2* is dominated by three high-risk regions—clusters 2, 3, and 8—located in the catalytic, anticodon, and tRNA-binding domains. These clusters show the strongest coupling between structural vulnerability and severe MLASA2 phenotypes, while other regions contribute variably to disease expressivity depending on their domain context, functional relevance, and recurrence across cases.

## 4. Discussion

This study provides a comprehensive integration of the clinical spectrum, survival, and pathogenic architecture of *YARS2* in subjects with MLASA2. The pooled meta-analysis confirms that anemia—predominantly of the sideroblastic phenotype—is the most common early presentation, followed by lactic acidosis and transfusion dependence. Although MLASA2 is classically described as a childhood-onset disorder, our analysis highlights marked heterogeneity: nearly two-thirds of patients develop symptoms before age five, with several cases presented in the first months of life, yet one-third manifest symptoms in adolescence or adulthood [6,7,25,26,27,28,29,30,31,32,33,34,35,36,37,38].

In most cases, diagnosis is not established early, and the estimated time delay in diagnosis exceeded 10 years in 44% of cases, consistent with the diagnostic odyssey that patients with genetic diseases and families typically experience due to the historically limited availability of molecular testing and limitations in the interpretation of potential PV [39].

This meta-analysis identified anemia as the most common clinical manifestation, present in 88.9% of cases, predominantly of the sideroblastic phenotype (86.1%), whereas lactic acidosis (76.5%) and transfusion-dependency (69.4%) were the second- and third-most common features, respectively.

Anemia was mostly diagnosed during the first ten years of life (68.1% of the cases), a period where the bone marrow is highly active and the demands on hematopoietic tissue are extraordinarily high, and the pediatric red blood cell production rates, measured per kilogram of body weight, are higher than in adults [40,41]. Thus, it is plausible that PV in *YARS2* affects hematopoiesis because bone marrow is metabolically active and rapidly proliferative. However, there are exceptional cases in which anemia and asthenia are the only clinical features. Riley et al. (2018) [32] reported two cases of mild to moderate anemia harboring a pathogenic variant in *YARS2* and evidence of iron overload (high transferrin saturation), but no other clinical manifestations, except for asthenia in a 22-year-old woman. It highlights the clinical heterogeneity of MLASA2 [32].

Lactic acidosis is identified by biochemical testing and is not easily clinically recognized. In fact, it appeared only during exercise in some patients, as observed by Riley [32] and Rawles [35] in two and one patient, respectively. For such a reason, its identification is not easy and should be suspected in the presence of fatigue, weakness, tachypnea, and muscle complaints [42].

Remarkably, although myopathy is a hallmark of MLASA2, it was only found in 65.7% of patients, and exercise intolerance was presented in 51.4%, which is a consistent finding since the latter may result from myopathy and lactic acidosis [31].

The present study provides, to our knowledge, the first survival analysis for MLASA2. Based on all available data, our study revealed that most of the affected individuals survive into adulthood, but mortality accelerates after the third decade, with a major drop around age 50, consistent with progressive, multisystem mitochondrial disease [2]. Furthermore, cardiomyopathy and diagnosis before 10 years of age were significant predictors of reduced survival, while lactic acidosis, respiratory insufficiency, and homozygosity for p.(Phe52Leu) showed borderline associations. This suggests that higher severity of disease, evidenced by early age at presentation, heart muscle damage, and metabolic abnormalities, negatively impacts survival.

From a biological perspective, PVs in *YARS2* impair mitochondrial translation, leading to defective synthesis of respiratory chain components and in turn oxidative phosphorylation failure [7]. This results in chronic energy deficiency, increased oxidative stress, and metabolic imbalance, which disproportionately affect tissues with high energetic demands such as skeletal muscle, cardiac muscle, and the hematopoietic system [43]. These mechanisms provide a unifying explanation for the characteristic combination of myopathy, cardiomyopathy, lactic acidosis, and sideroblastic anemia observed in MLASA2, consistent with established models of mitochondrial multisystem disease [44].

The ethnic and geographic distribution of the cases revealed several important features. Firstly, European ancestry was the population with the highest number of MLASA2 cases (57.1%), and most cases were found among European settlers in Australia, as well as subjects from the UK, Italy, Spain, and European Americans. Second, 31.4% of cases are of Lebanese descent, even when reported cases came from multiple countries [8,27,28,29,33]. Third, no reports were identified among East Asian, African, or Latin American populations.

A major contribution of this study is the integration of clinical findings with a three-dimensional structural map of all reported *YARS2* PVs. We identified nine spatially coherent variant clusters, demonstrating that pathogenic variation in *YARS2* is not randomly distributed but instead concentrates in discrete structural regions across the catalytic, anticodon-binding, and tRNA-binding domains. This structural organization provides a mechanistic framework to interpret genotype–phenotype relationships.

Among all clusters, Cluster 3 stands out as the major pathogenic region, characterized by its large size, high representation of affected individuals, and concentration of PVs within the catalytic domain of *YARS2*. The convergence of multiple damaging in silico predictions and the consistent association with multisystem phenotypes support the biological relevance of this region in MLASA2 pathogenesis. However, it is important to recognize that all cases harboring the p.(Phe52Leu) variant, which belong to Cluster 3, were found exclusively in subjects of Lebanese descent. This observation raises the possibility that the enrichment of PVs in this cluster is influenced by a founder effect rather than solely reflecting intrinsic structural vulnerability. Since Cluster 3 also encompasses several additional PV affecting distinct residues across the catalytic core [p.(Gly191Asp), p.(Gly191Val), p.(Ser203Ile), p.(Gly244Ala), p.(Thr197_Leu208del), p.(Met195Ile), p.(Leu61Val), p.(Leu208Arg), and p.(Pro122Arg), this cluster may represent a true functional hotspot where both population-specific recurrence and protein-level susceptibility converge. Furthermore, the moderate mortality rate observed in this cluster (22%) despite several clinical manifestations further indicates that disruption of the catalytic core is compatible with survival into adulthood while causing significant mitochondrial dysfunction.

Two additional Clusters (2 and 8) showed a pattern of extreme clinical severity despite small sample sizes. This combination of high penetrance of multisystem involvement, severe metabolic phenotypes, and high mortality (50% in cluster 2 and 67% in Cluster 8) may reflect the deleterious nature of PVs affecting these regions. The strong in silico pathogenicity signals and marked ΔΔG-based structural destabilization in both clusters further support this interpretation. Cluster 2, located within a tightly packed catalytic microdomain, and Cluster 8, embedded in the tRNA-binding interface, are predicted to disrupt essential steps of the aminoacylation cycle, suggesting that these regions could be important for cell mitochondrial function. Their classification as high-severity, high-evidence microdomains underscores that pathogenic potential, rather than case count alone, may be useful in guiding hotspot designation in ultra-rare disorders. Nevertheless, it is important to acknowledge that the small number of MLASA2 cases harboring PVs in Clusters 2 and 8 may lead to an overestimation of their pathogenic impact, or alternatively, may reflect PVs that are truly lethal and therefore underrepresented among surviving individuals.

Cluster 6, located in the anticodon-binding domain, represents a clinically informative region. Although no mortality was observed, the consistent presence of myopathy, transfusion dependence, and lactic acidosis across affected individuals, together with recurrence in five patients, indicates that impaired residues in this region contribute substantially to MLASA2 pathogenesis. Compared with Clusters 2, 3, and 8, Cluster 6 appears to disrupt mitochondrial translation sufficiently to cause multisystem disease without early lethality, potentially reflecting the low frequency of cardiomyopathy observed in this group.

Clusters 1 and 5 showed moderate pathogenicity with reproducible but heterogeneous phenotypes, suggesting partial loss-of-function or tissue-limited effects, notably without cardiomyopathy in Cluster 1 or transfusion dependence in Cluster 5. In contrast, Clusters 4, 7, and 9 (recurrent in only two cases) were associated with low clinical severity and limited supporting evidence, consistent with structurally tolerant regions. Together, these findings highlight the importance of integrating structural clustering with phenotypic and in silico data rather than relying solely on structural proximity.

Collectively, three-dimensional structural analysis, hotspot cluster identification, and their clinical correlations suggest that MLASA2 pathogenicity is driven by a small number of structurally critical microdomains—located in a few regions of the catalytic, anticodon-binding, and tRNA-binding domains—while other areas of *YARS2* remain comparatively resilient to variation. Our approach to cluster evaluation not only enhances biological plausibility but also provides a practical framework for interpreting newly identified *YARS2* PVs in clinical sequencing, particularly when they fall within high-risk regions where disease penetrance and lethality are greatest or within clusters of silent or mild phenotypic expression. For example, although no MLASA2 cases have yet been reported with PVs affecting residues 78, 79, or 80, these amino acids fall within structural hotspot Cluster 3. Consequently, a future missense variant involving any of these residues would localize to a functionally constrained catalytic region enriched for PVs, thereby supporting an increased prior probability of pathogenicity. In such cases, cluster membership may strengthen diagnostic confidence when integrated with established criteria, including population rarity, deleterious in silico predictions, and phenotypic concordance. Nevertheless, this framework requires further validation and is intended to complement current variant interpretation guidelines.

We acknowledge several limitations of this study. Primary clinical data were incomplete for a subset of patients included in the survival analysis, and follow-up duration was restricted to that reported in the primary articles, potentially limiting the accuracy of long-term outcome estimates. In addition, reliance on case reports and small case series introduces an inherent reporting bias toward more severe or clinically distinctive phenotypes, while individuals with milder disease may be underrepresented. Conversely, survivor bias may also influence survival estimates, as patients with early lethality may be less likely to be reported or to have extended follow-up, potentially leading to an overestimation of long-term survival. The structural hotspot analysis is further constrained by small sample sizes—particularly in highly severe clusters such as 2 and 8, where high mortality may reflect true biological lethality but also selective survival among reported cases. Additionally, functional predictions and ΔΔG estimates are derived from in silico modeling rather than experimental assays. Finally, the predominance of cases from European and Lebanese ancestry limits global generalizability and may obscure population-specific variation. Despite these constraints, the integrative analysis of clinical, survival, and structural data provides a coherent and biologically plausible framework for understanding *YARS2* pathogenicity and other genetic diseases in the future.

## 5. Conclusions

This study broadens the clinical and molecular spectrum of MLASA2 by integrating the pooled prevalence of clinical phenotypes, survival patterns, prognostic factors, and the 3D structural architecture of *YARS2*. Anemia- particularly the sideroblastic phenotype- and lactic acidosis were the most prevalent, whereas myopathy, exercise intolerance, and cardiomyopathy are much less common and vary widely across patients. Survival declines notably after the third decade, particularly among individuals with cardiomyopathy or early-onset disease, underscoring the clinical relevance of timely recognition. The uneven geographic distribution of cases suggests underdiagnosis or true population differences driven by founder effects and ancestry-related allele frequencies in some regions. By mapping Mt-TyrRS PVs onto structurally coherent 3D *YARS2* microdomains, we show that MLASA2 pathogenicity arises from the disruption of a few specific regions essential for anticodon recognition, tRNA (Tyr) aminoacylation, and tRNA binding. This framework not only will facilitate the interpretation of newly identified *YARS2* PVs but also provides a scalable strategy for analyzing structural–clinical relationships in other rare genetic diseases.

## Figures and Tables

**Table 1:** General characteristics of the included studies in the systematic review and meta-analysis.

Study	Country	Ascendence	Method of PV Identification	Screened Patients	Num. Affected Patients	PV Reported
Rawles, 1974 [35]	UK	2 Scottish	TSeq	2	2	p.(Leu392Ser) n.d.
Riley, 2010 [7]	Australia	Lebanese	Microarrays	3	3	p.(Phe52Leu) p.(Phe52Leu) p.(Phe52Leu)
Sasarman, 2012 [28]	Canada	Lebanese	RT-PCR	1	1	p.(Gly46Asp)
Riley, 2013 [27]	Australia	2 Lebanese, 1 French	Sanger	12	3	p.(Phe52Leu) p.(Phe52Leu) p.[Gly191Asp];[Arg360*]
Shahni, 2013 [26]	UK	Lebanese	Long range PCR, and Sanger	1	1	p.(Phe52Leu)
Ardissone, 2014 [29]	Italy	Italian	Sanger and Southern blot	2	2	p.(Asp311Glu) p.(Asp311Glu)
Nakajima, 2014 [30]	Turkey	Türkiye	WES	1	1	p.(Ser435Gly)
Sommerville, 2017 [31]	UK	2 Scottish 2 Jordanian	Sanger and WES	4	4	p.(Leu392Ser) p.[Cys369Tyr];[Val383_Glu388dup] p.(Gly46Asp) n.d.
Riley, 2018 [32]	USA	4 Lebanese, Caucasian Dutch, Caucasian American, African American, Spanish	TSeq and WES	14	14	p.(Asp311Glu) p.[Ser203Ile];p[c.1104-1G>A] p.[Ser33*];p.[Tyr236Cys] p.[Ser33*];p.[Tyr236Cys] p.[Gly191Val];p.[Ile454Serfs*10] p.[Gly191Val];p.[Ile454Serfs*10] p.[Gly191Val, Gly244Ala];p.[Asp311Glu] p.[Gly191Val];p.[Thr197_Leu208del] p.[Met195Ile];p.[Leu389Cysfs*6] p.(Leu61Val) p.(Phe52Leu) p.(Phe52Leu) p.(Phe52Leu) p.(Phe52Leu)
Smith, 2018 [34]	UK	Caucasian	NGS	1	1	p.[Pro122Arg];[Leu208Arg]
Carreño-Gago, 2021 [33]	Spain	Spanish	WES	2	2	p.[Gly105Alafs*4];[p.Ile464Thr)] p.[Gly105Alafs*4];[p.Ile464Thr)]
Rudacks, 2022 [25]	Australia	Australian	WES	1	1	p.[Ser33*];[Arg316Ser]
Villafán-Bernal, 2025 [36]	Mexico	Mexican mestizo	WES	1	1	p.(Asp311Glu)

n.d.: not determined; PV: pathogenic variant; RT-PCR: Reverse Transcriptase–Polymerase Chain Reaction; WES: Whole-genome sequencing; NGS: Next-generation sequencing; TSeq: Targeted sequencing.

**Table 2:** Estimated survival at 5, 10, 20, 30, 40, and 50 years of age.

Age	Patients at Risk	Events	Cumulative Survival	Standard Error	Lower 95% IC	Upper 95% IC
5	29	2	0.941	0.0404	0.865	1.000
10	25	0	0.941	0.0404	0.865	1.000
20	17	2	0.857	0.0679	0.734	1.000
30	10	2	0.707	0.1117	0.519	0.964
40	8	0	0.707	0.1117	0.519	0.964
50	3	2	0.424	0.1688	0.195	0.925

**Table 3:** Structural Evaluation of Mt-TyrRS Hotspot Clusters and its Clinical Correlations.

Cluster Number	Num. of Residues	Residues	Domain(s)	Mean Intra-Cluster Distance (Å)	Cr1	Cr2 ^&^	Cr3 ^&^	Cr4 ^&^	Cr5	Cr6	CSS	NAS by Cluster *	Clinical Manifestations	Global Hotspot Classification
1	11	32, 33, 34, 35, 36, 67, 68, 69, 104, 105, 106	Catalytic	7.13	Yes	No	No	No	No	4	0.42	4	Myopathy: 50%. Cardiomyopathy: 0%. Lactic acidosis: 67%. Transfusion dependence: 50%. Median age: 27.0. Mortality rate: 25%.	Clinical: Medium; Strength evidence of pathogenicity: Medium
2	3	45, 46, 47	Catalytic	4.35	Yes	Yes	No	Yes	Yes	2	1	2	Myopathy: 100%. Cardiomyopathy: 100%. Lactic acidosis: 100%. Transfusion dependence: 100%. Median age: 32.0. Mortality rate: 50%.	Clinical: High; Strength evidence of pathogenicity: High
3	44	51, 52, 53, 60, 61, 62, 78, 79, 80, 121, 122, 123, 178, 179, 180, 184, 185, 186, 190, 191, 192, 194, 195, 196, 197, 198, 199, 200, 201, 202, 203, 204, 205, 206, 207, 208, 209, 210, 243, 244, 245, 250, 251, 252	Catalytic	7.95	Yes	No	Yes	Yes	Yes	17	0.63	17	Myopathy: 65%. Cardiomyopathy: 47%. Lactic acidosis: 75%. Transfusion dependence: 67%. Median age: 21.5. Mortality rate: 22%.	Clinical: High, Strength evidence of pathogenicity: High
4	3	235, 236, 237	Catalytic	4.6	Yes	No	No	Yes	No	2	0.13	2	Myopathy: 0%. Cardiomyopathy: 0%. Lactic acidosis: 0%. Transfusion dependence: 50%. Median age: 5.0. Mortality rate: 0%.	Clinical: Low; Strength evidence of pathogenicity: Low
5	18	294, 295, 296, 368, 369, 370, 382, 383, 384, 385, 386, 387, 388, 389, 390, 391, 392, 393	Anticodon binding; Catalytic; tRNAb	7.83	Yes	No	Yes	No	Yes	2	0.5	2	Myopathy: 100%. Cardiomyopathy: 50%. Lactic acidosis: 50%. Transfusion dependence: 0%. Median age: 60.0. Mortality rate: 50%.	Clinical: Medium; Strength evidence of pathogenicity: Medium
6	5	309, 310, 311, 312, 313	Anticodon binding	5.42	Yes	No	No	Yes	No	5	0.78	5	Myopathy: 100%. Cardiomyopathy: 33%. Lactic acidosis: 80%. Transfusion dependence: 100%. Median age: 13.0. Mortality rate: 0%.	Clinical: High; Strength evidence of pathogenicity: Medium
7	3	359, 360, 361	Anticodon binding	4.42	Yes	No	No	No	No	0	0	0	Myopathy: NA. Cardiomyopathy: NA. Lactic acidosis: NA. Transfusion dependence: NA. Median age: NA. Event rate: NA.	Clinical: Low; Strength evidence of pathogenicity: Low
8	8	434, 435, 436, 463, 464, 465, 466, 467	tRNAb	6.76	Yes	Yes	No	Yes	Yes	3	0.92	3	Myopathy: 100%. Cardiomyopathy: 100%. Lactic acidosis: 100%. Transfusion dependence: 67%. Median age: 48.0. Mortality rate: 67%.	Clinical: High; Strength evidence of pathogenicity: High
9	3	453, 454, 455	tRNAb	4.72	Yes	No	No	No	No	0	0	0	Myopathy: NA. Cardiomyopathy: NA. Lactic acidosis: NA. Transfusion dependence: NA. Median age: NA. Event rate: NA.	Clinical: Low; Strength evidence of pathogenicity: Low

Cr1: Structural cluster (≥3 residues); Cr2: REVEL enriched (≥50% ≥0.5); Cr3: Alpha Missense enriched (≥50% P); Cr4: ESM1b enriched (≥50% D); Cr5: High ΔΔG (≥1 variant |ΔΔG| ≥ 0.5); Cr6: Number of cases reported in the literature and CSS; * NAS, Number of affected subjects with PVs for each cluster. ^&^ Detailed scores for Alpha Missense, REVEL, ESM1b, and 40 other computational predictors are in Supplementary File S1. Cr = criteria.

## Data Availability

No new data were created or analyzed in this study. Data sharing is not applicable to this article.

## Supplementary Materials

The following supporting information can be downloaded at https://www.mdpi.com/article/10.3390/cimb48010095/s1.

## Abbreviations

The following abbreviations are used in this manuscript:

**Abbreviation**	**Meaning**
ATP6	Mitochondrially encoded ATP synthase membrane subunit 6
CIs	Confidence Intervals
DNA	Deoxyribonucleic Acid
I^2^	I-squared (measure of heterogeneity in meta-analysis)
INMEGEN	Instituto Nacional de Medicina Genómica
JBI	Joanna Briggs Institute
MLASA	Myopathy, Lactic Acidosis and Sideroblastic Anemia
MLASA2	Myopathy, Lactic Acidosis and Sideroblastic Anemia Type 2
Mt-TyrRS	Mitochondrial Tyrosyl-tRNA Synthetase
NGS	Next-Generation Sequencing
n.d.	Not Determined
OMIM	Online Mendelian Inheritance in Man
PCR	Polymerase Chain Reaction
PLOGIT	Logit transformation used in meta-analyses
PRISMA	Preferred Reporting Items for Systematic Reviews and Meta-Analyses
PROSPERO	Preferred Reporting Items for Systematic reviews and Meta-Analyses
PFT	Freeman–Tukey double arcsine transformation
PUS1	Pseudouridine Synthase 1 gene
PV	Pathogenic Variant
REM	Random-Effects Model
RT-PCR	Reverse Transcriptase–Polymerase Chain Reaction
tRNA	Transfer Ribonucleic Acid
TyrRS	Tyrosyl-tRNA Synthetase
WES	Whole Exome Sequencing
WGS	Whole-Genome Sequencing

## Appendix A

### Appendix A.1. Query for Each Database

PubMed Query: 94 results (*YARS2* OR MLASA2) OR (myopathy lactic acidosis anemia)
Scopus Query: 116 results (*YARS2* OR MLASA2) OR (myopathy lactic acidosis anemia)
Web of Science Query: 288 results (*YARS2* OR MLASA2) OR (myopathy lactic acidosis anemia)
Total studies identified: n = 498

### Appendix A.2. Evaluation of Study Quality

The Joanna Bridge Institute (JBI) critical appraisal tools for case reports and case series were employed for evaluation of the study quality.

**Table A1:** Case Series.

Study	Q1	Q2	Q3	Q4	Q5	Q6	Q7	Q8	Q9	Q10	TOTAL
Rawles et al. (1974) [35]	Yes	Yes	Yes	Yes	Yes	Yes	Yes	Yes	Unclear	Unclear	8
Riley et al. (2010) [7]	Yes	Yes	Yes	Yes	Yes	Yes	Yes	Yes	Unclear	Unclear	8
Riley et al. (2013) [27]	Yes	Yes	Yes	Yes	Yes	Yes	Yes	Yes	Unclear	Yes	9
Riley et al. (2018) [32]	Yes	Yes	Yes	Yes	Yes	Yes	Yes	Yes	Unclear	Yes	9
Ardissone et al. (2014) [29]	Yes	Yes	Yes	Yes	Yes	Yes	Yes	Yes	Unclear	Unclear	8
Sommerville et al. (2017) [31]	Yes	Yes	Yes	Yes	Yes	Yes	Yes	Yes	Unclear	Yes	9
Carreño-Gago et al. (2021) [33]	Yes	Yes	Yes	Yes	Yes	Yes	Yes	Yes	Unclear	Yes	9

Q1. Were there clear criteria for inclusion in the case series? Q2. Was the condition measured in a standard, reliable way for all participants included in the case series? Q3. Were valid methods used for identification of the condition for all participants included in the case series? Q4. Did the case series have consecutive inclusion of participants? Q5. Did the case series have complete inclusion of participants? Q6. Was there clear reporting of the demographics of the participants in the study? Q7. Was there clear reporting of clinical information of the participants? Q8. Were the outcomes or follow up results of cases clearly reported? Q9. Was there clear reporting of the presenting site(s)/clinic(s) demographic information? Q10. Was statistical analysis appropriate.

**Table A2:** Case Reports.

Study	Q1	Q2	Q3	Q4	Q5	Q6	Q7	Q8	TOTAL
Rudaks et al. (2022) [25]	Yes	Yes	Yes	Yes	Yes	Yes	Yes	Yes	8
Sasarman et al. (2012) [28]	Yes	Yes	Yes	Yes	Yes	Yes	No	Yes	7
Shahni et al. (2013) [26]	Yes	Yes	Yes	Yes	Yes	Yes	No	Yes	7
Nakajima et al. (2014) [30]	Yes	Yes	Yes	Yes	Yes	Yes	No	Yes	7
Smith et al. (2018) [34]	Yes	Yes	Yes	Yes	Yes	Yes	No	Yes	7
Villafán-Bernal et al., (2025) [36]	Yes	Yes	Yes	Yes	Yes	Yes	Yes	Yes	8

Q1. Were patient’s demographic characteristics clearly described? Q2. Was the patient’s history clearly described and presented as a timeline? Q3. Was the current clinical condition of the patient on presentation clearly described? Q4. Were diagnostic tests or assessment methods and the results clearly described? Q5. Was the intervention(s) or treatment procedure(s) clearly described? Q6. Was the post-intervention clinical condition clearly described? Q7. Were adverse events (harms) or unanticipated events identified and described? Q8. Does the case report provide takeaway lessons?
