# Supplementary material for: Correlation of MLASA2 Clinical Phenotype and Survival with Mt-TyrRS Protein Damage: Linking Systematic Review, Meta-Analysis and 3D Hotspot Mapping
Source: Curr Issues Mol Biol. 2026 Jan 16;48(1):95. doi: 10.3390/cimb48010095 (PMC12839713; doi:10.3390/cimb48010095)
Supplement: Supplementary file 1 [file cimb-48-00095-s001.zip › SUPPLEMENTARY FILE 1. MLASA2-YARS2.pdf]

Supplementary file S1. Sensitivity analysis

Study	Pooled	Lower bound CI	Cpper bound CI	I^2	K included	Manifestation
Rudacks, 2022	-0.993053546561137	-1.71813573107524	-0.267971362047035	0	10	failuretothrive
Riley, 2010	-1.22708638661809	-1.81706793198949	-0.637104841246691	0	10	failuretothrive
Sasarmanm, 2012	-0.993053546561137	-1.71813573107524	-0.267971362047035	0	10	failuretothrive
Riley, 2013	-1.07243951349336	-1.81424733634456	-0.33063169064215	0	10	failuretothrive
Shahni, 2013	-1.11501402970843	-1.73109670632282	-0.498931353094048	0	10	failuretothrive
Ardissone, 2014	-0.960698311757619	-1.67858375370513	-0.242812869810106	0	10	failuretothrive
Nakajima, 2014	-0.993053546561137	-1.71813573107524	-0.267971362047035	0	10	failuretothrive
Sommerville, 2017	-0.889519910402662	-1.54465689774271	-0.234382923062618	0	10	failuretothrive
Riley, 2018	-0.74649183983614	-1.50834574545248	0.0153620657801972	0	10	failuretothrive
Smith, 2018	-0.993053546561137	-1.71813573107524	-0.267971362047035	0	10	failuretothrive
Carreño-Gago, 2021	-0.960698311757619	-1.67858375370513	-0.242812869810106	0	10	failuretothrive
Rudacks, 2022	-1.39252215521674	-1.94918536984533	-0.83585894058814	0	10	nystagmus
Riley, 2010	-1.32614294792763	-1.87398873909847	-0.77829715675678	0	10	nystagmus
Sasarmanm, 2012	-1.39252215521674	-1.94918536984533	-0.83585894058814	0	10	nystagmus
Riley, 2013	-1.54152659016898	-2.04266812622754	-1.04038505411042	0	10	nystagmus
Shahni, 2013	-1.39252215521674	-1.94918536984533	-0.83585894058814	0	10	nystagmus
Ardissone, 2014	-1.35487646196631	-1.91421159454874	-0.795541329383892	0	10	nystagmus
Nakajima, 2014	-1.39252215521674	-1.94918536984533	-0.83585894058814	0	10	nystagmus
Sommerville, 2017	-1.39245051064239	-1.99838644847519	-0.786514572809584	0	10	nystagmus
Riley, 2018	-1.19018285206757	-1.49481114620046	-0.885554557934676	0	10	nystagmus
Smith, 2018	-1.39252215521674	-1.94918536984533	-0.83585894058814	0	10	nystagmus
Carreño-Gago, 2021	-1.35487646196631	-1.91421159454874	-0.795541329383892	0	10	nystagmus
Rudacks, 2022	-1.21536965731584	-1.98296596004499	-0.447773354586693	0	10	ptosis
Riley, 2010	-0.988615549392866	-1.86021382414644	-0.117017274639292	0	10	ptosis

**Sasarmanm, 2012**	-1.05987072632035	-1.94889915371917	-0.170842298921542	0	10	ptosis
**Riley, 2013**	-0.988615549392866	-1.86021382414644	-0.117017274639292	0	10	ptosis
**Shahni, 2013**	-1.21536965731584	-1.98296596004499	-0.447773354586693	0	10	ptosis
**Ardissone, 2014**	-1.01907703904674	-1.90346364866124	-0.134690429432243	0	10	ptosis
**Nakajima, 2014**	-1.05987072632035	-1.94889915371917	-0.170842298921542	0	10	ptosis
**Sommerville, 2017**	-1.25001038529405	-2.22944186251287	-0.270578908075238	0	10	ptosis
**Riley, 2018**	-0.847803983212807	-1.55452647957263	-0.14108148685298	0	10	ptosis
**Smith, 2018**	-1.05987072632035	-1.94889915371917	-0.170842298921542	0	10	ptosis
**Carreño-Gago, 2021**	-1.01907703904674	-1.90346364866124	-0.134690429432243	0	10	ptosis
**Rudacks, 2022**	-1.80709090185215	-2.36136783241366	-1.25281397129064	0	10	ocular_albinism
**Riley, 2010**	-1.52606354156102	-2.36608065707011	-0.686046426051927	0	10	ocular_albinism
**Sasarmanm, 2012**	-1.60883087840302	-2.44087571620068	-0.776786040605356	0	10	ocular_albinism
**Riley, 2013**	-1.52606354156102	-2.36608065707011	-0.686046426051927	0	10	ocular_albinism
**Shahni, 2013**	-1.60883087840302	-2.44087571620068	-0.776786040605356	0	10	ocular_albinism
**Ardissone, 2014**	-1.56226510361211	-2.40581003349267	-0.718720173731556	0	10	ocular_albinism
**Nakajima, 2014**	-1.60883087840302	-2.44087571620068	-0.776786040605356	0	10	ocular_albinism
**Sommerville, 2017**	-1.45262330738448	-2.2575642543197	-0.647682360449265	0	10	ocular_albinism
**Riley, 2018**	-1.35157922366497	-2.04220336511057	-0.660955082219362	0	10	ocular_albinism
**Smith, 2018**	-1.60883087840302	-2.44087571620068	-0.776786040605356	0	10	ocular_albinism
**Carreño-Gago, 2021**	-1.56226510361211	-2.40581003349267	-0.718720173731556	0	10	ocular_albinism
**Rudacks, 2022**	-1.80709090185215	-2.36136783241366	-1.25281397129064	0	10	opticnerve_hypoplasia
**Riley, 2010**	-1.52606354156102	-2.36608065707011	-0.686046426051927	0	10	opticnerve_hypoplasia
**Sasarmanm, 2012**	-1.60883087840302	-2.44087571620068	-0.776786040605356	0	10	opticnerve_hypoplasia
**Riley, 2013**	-1.52606354156102	-2.36608065707011	-0.686046426051927	0	10	opticnerve_hypoplasia

Shahni, 2013	-1.60883087840302	-2.44087571620068	-0.776786040605356	0	10	opticnerve_hypoplasia
Ardissone, 2014	-1.56226510361211	-2.40581003349267	-0.718720173731556	0	10	opticnerve_hypoplasia
Nakajima, 2014	-1.60883087840302	-2.44087571620068	-0.776786040605356	0	10	opticnerve_hypoplasia
Sommerville, 2017	-1.45262330738448	-2.2575642543197	-0.647682360449265	0	10	opticnerve_hypoplasia
Riley, 2018	-1.35157922366497	-2.04220336511057	-0.660955082219362	0	10	opticnerve_hypoplasia
Smith, 2018	-1.60883087840302	-2.44087571620068	-0.776786040605356	0	10	opticnerve_hypoplasia
Carreño-Gago, 2021	-1.56226510361211	-2.40581003349267	-0.718720173731556	0	10	opticnerve_hypoplasia
Rudacks, 2022	-1.39252215521674	-1.94918536984533	-0.83585894058814	0	10	strabismus
Riley, 2010	-1.17090559414028	-1.89239187483112	-0.449419313449432	0	10	strabismus
Sasarmanm, 2012	-1.2390914875464	-1.97356795037811	-0.504615024714676	0	10	strabismus
Riley, 2013	-1.37014470534518	-2.10149696812436	-0.638792442566006	0	10	strabismus
Shahni, 2013	-1.2390914875464	-1.97356795037812	-0.504615024714676	0	10	strabismus
Ardissone, 2014	-1.20024604600096	-1.93339506282358	-0.467097029178343	0	10	strabismus
Nakajima, 2014	-1.2390914875464	-1.97356795037812	-0.504615024714676	0	10	strabismus
Sommerville, 2017	-1.2120214851312	-2.00848482864706	-0.415558141615342	0	10	strabismus
Riley, 2018	-1.0335933177099	-1.57284705411533	-0.49433958130446	0	10	strabismus
Smith, 2018	-1.2390914875464	-1.97356795037812	-0.504615024714676	0	10	strabismus
Carreño-Gago, 2021	-1.20024604600096	-1.93339506282358	-0.467097029178344	0	10	strabismus
Rudacks, 2022	-1.53303695661052	-2.18332408779474	-0.882749825426302	0	10	vocalcord_paralysis
Riley, 2010	-1.72735065566918	-2.30464844958442	-1.15005286175394	0	10	vocalcord_paralysis
Sasarmanm, 2012	-1.53303695661052	-2.18332408779474	-0.882749825426302	0	10	vocalcord_paralysis
Riley, 2013	-1.45824761654953	-2.1101490957232	-0.806346137375861	0	10	vocalcord_paralysis
Shahni, 2013	-1.53303695661052	-2.18332408779474	-0.882749825426302	0	10	vocalcord_paralysis
Ardissone, 2014	-1.49083259517086	-2.14969542862659	-0.831969761715138	0	10	vocalcord_paralysis
Nakajima, 2014	-1.53303695661052	-2.18332408779474	-0.882749825426302	0	10	vocalcord_paralysis

**Sommerville, 2017**	-1.39245051064239	-1.99838644847519	-0.786514572809584	0	10	vocalcord_paralysis
**Riley, 2018**	-1.30226395320934	-1.75854382353791	-0.845984082880773	0	10	vocalcord_paralysis
**Smith, 2018**	-1.53303695661052	-2.18332408779474	-0.882749825426302	0	10	vocalcord_paralysis
**Carreño-Gago, 2021**	-1.49083259517086	-2.14969542862659	-0.831969761715138	0	10	vocalcord_paralysis
**Rudacks, 2022**	-1.53303695661052	-2.18332408779474	-0.882749825426302	0	10	epilepsy
**Riley, 2010**	-1.45824761654953	-2.1101490957232	-0.806346137375862	0	10	epilepsy
**Sasarmanm, 2012**	-1.53303695661052	-2.18332408779474	-0.882749825426302	0	10	epilepsy
**Riley, 2013**	-1.72735065566918	-2.30464844958442	-1.15005286175394	0	10	epilepsy
**Shahni, 2013**	-1.53303695661052	-2.18332408779474	-0.882749825426303	0	10	epilepsy
**Ardissone, 2014**	-1.49083259517086	-2.14969542862659	-0.831969761715138	0	10	epilepsy
**Nakajima, 2014**	-1.53303695661052	-2.18332408779474	-0.882749825426303	0	10	epilepsy
**Sommerville, 2017**	-1.39245051064239	-1.99838644847519	-0.786514572809584	0	10	epilepsy
**Riley, 2018**	-1.30226395320934	-1.75854382353791	-0.845984082880773	0	10	epilepsy
**Smith, 2018**	-1.53303695661052	-2.18332408779474	-0.882749825426302	0	10	epilepsy
**Carreño-Gago, 2021**	-1.49083259517086	-2.14969542862659	-0.831969761715138	0	10	epilepsy
**Rudacks, 2022**	-1.53303695661052	-2.18332408779474	-0.882749825426302	0	10	hypoplasia_corpuscall
**Riley, 2010**	-1.45824761654953	-2.1101490957232	-0.806346137375862	0	10	hypoplasia_corpuscall
**Sasarmanm, 2012**	-1.53303695661052	-2.18332408779474	-0.882749825426302	0	10	hypoplasia_corpuscall
**Riley, 2013**	-1.72735065566918	-2.30464844958442	-1.15005286175394	0	10	hypoplasia_corpuscall
**Shahni, 2013**	-1.53303695661052	-2.18332408779474	-0.882749825426303	0	10	hypoplasia_corpuscall
**Ardissone, 2014**	-1.49083259517086	-2.14969542862659	-0.831969761715138	0	10	hypoplasia_corpuscall
**Nakajima, 2014**	-1.53303695661052	-2.18332408779474	-0.882749825426303	0	10	hypoplasia_corpuscall
**Sommerville, 2017**	-1.39245051064239	-1.99838644847519	-0.786514572809584	0	10	hypoplasia_corpuscall
**Riley, 2018**	-1.30226395320934	-1.75854382353791	-0.845984082880773	0	10	hypoplasia_corpuscall
**Smith, 2018**	-1.53303695661052	-2.18332408779474	-0.882749825426302	0	10	hypoplasia_corpuscall

Carreño-Gago, 2021	-1.49083259517086	-2.14969542862659	-0.831969761715138	0	10	hypoplasia_corpuscall
Rudacks, 2022	0.415436643668058	-0.427177518291964	1.25805080562808	0	10	myopathy
Riley, 2010	0.42695793835243	-0.366137165013061	1.22005304171792	0	10	myopathy
Sasarmanm, 2012	0.415436643668058	-0.427177518291964	1.25805080562808	0	10	myopathy
Riley, 2013	0.285588503256634	-0.485931488206168	1.05710849471944	0	10	myopathy
Shahni, 2013	0.415436643668058	-0.427177518291964	1.25805080562808	0	10	myopathy
Ardissone, 2014	0.627831431919373	-0.122485445991756	1.3781483098305	0	10	myopathy
Nakajima, 2014	0.415436643668058	-0.427177518291964	1.25805080562808	0	10	myopathy
Sommerville, 2017	0.185952235007732	-0.494223779772676	0.86612824978814	0	10	myopathy
Riley, 2018	0.79672549872366	-0.104128975470253	1.69757997291757	0	10	myopathy
Smith, 2018	0.415436643668059	-0.427177518291964	1.25805080562808	0	10	myopathy
Carreño-Gago, 2021	0.345230427546735	-0.464362265875488	1.15482312096896	0	10	myopathy
Rudacks, 2022	-0.474404632890304	-1.35697099026863	0.408161724488023	0.0839460667382939	10	hmyocardiopathy
Riley, 2010	-0.353772314031179	-1.31440368104433	0.606859052981973	0.159718958431453	10	hmyocardiopathy
Sasarmanm, 2012	-0.474404632890304	-1.35697099026863	0.408161724488023	0.0839460667382939	10	hmyocardiopathy
Riley, 2013	-0.353772314031179	-1.31440368104433	0.606859052981973	0.159718958431453	10	hmyocardiopathy
Shahni, 2013	-0.474404632890304	-1.35697099026863	0.408161724488023	0.0839460667382939	10	hmyocardiopathy
Ardissone, 2014	-0.2948253074457	-1.19132617028925	0.60167555539785	0.107701634887373	10	hmyocardiopathy
Nakajima, 2014	-0.474404632890304	-1.35697099026863	0.408161724488023	0.0839460667382939	10	hmyocardiopathy
Sommerville, 2017	-0.624017554943577	-1.54080042813229	0.292765318245134	0	10	hmyocardiopathy
Riley, 2018	-0.0191262087934772	-0.7275440011265	0.689291583539545	0	10	hmyocardiopathy
Smith, 2018	-0.333024732090117	-1.25108531822463	0.585035854044396	0.145636558820019	10	hmyocardiopathy
Carreño-Gago, 2021	-0.2948253074457	-1.19132617028925	0.60167555539785	0.107701634887373	10	hmyocardiopathy
Rudacks, 2022	0.328600765688369	-0.874860704001086	1.53206223537782	0.416202931471409	10	muscleweakness

**Riley, 2010**	0.230385032146509	-0.933728928554813	1.39449899284783	0.367871481860872	10	muscleweakness
**Sasarmanm, 2012**	0.510652537337895	-0.656344334198535	1.67764940887433	0.40712824155432	10	muscleweakness
**Riley, 2013**	0.383735782259792	-0.859065589024522	1.62653715354411	0.424208565592554	10	muscleweakness
**Shahni, 2013**	0.328600765688369	-0.874860704001085	1.53206223537782	0.416202931471409	10	muscleweakness
**Ardissone, 2014**	0.557902299525265	-0.568981981840451	1.68478658089098	0.376125002371864	10	muscleweakness
**Nakajima, 2014**	0.328600765688369	-0.874860704001086	1.53206223537782	0.416202931471409	10	muscleweakness
**Sommerville, 2017**	0.145346399554424	-0.961917857120873	1.25261065622972	0.297474676135696	10	muscleweakness
**Riley, 2018**	0.826156026359924	-0.0594970668642539	1.7118091195841	0	10	muscleweakness
**Smith, 2018**	0.328600765688369	-0.874860704001086	1.53206223537782	0.416202931471409	10	muscleweakness
**Carreño-Gago, 2021**	0.272091024893771	-0.912744427447125	1.45692647723467	0.392424831120649	10	muscleweakness
**Rudacks, 2022**	0.170487793311046	-1.01070378280316	1.35167936942525	0.353259475345972	10	exerciseintolerance
**Riley, 2010**	-0.138460759641771	-1.26027289296834	0.9833513736848	0.248970070054137	10	exerciseintolerance
**Sasarmanm, 2012**	0.170487793311046	-1.01070378280316	1.35167936942525	0.353259475345972	10	exerciseintolerance
**Riley, 2013**	0.243492744747272	-0.872113623864141	1.35909911335868	0.299253679405579	10	exerciseintolerance
**Shahni, 2013**	-0.0192299693369316	-1.20569512981044	1.16723519113658	0.333525940196094	10	exerciseintolerance
**Ardissone, 2014**	0.216077619169564	-0.931625790281445	1.36378102862057	0.327035191960542	10	exerciseintolerance
**Nakajima, 2014**	-0.0192299693369316	-1.20569512981044	1.16723519113658	0.333525940196094	10	exerciseintolerance
**Sommerville, 2017**	-0.255385167009992	-1.28759016738095	0.776819833360965	0.126107059717078	10	exerciseintolerance
**Riley, 2018**	0.391302273982549	-0.799351127406852	1.58195567537195	0.113706071849802	10	exerciseintolerance
**Smith, 2018**	-0.0192299693369316	-1.20569512981044	1.16723519113658	0.333525940196094	10	exerciseintolerance
**Carreño-Gago, 2021**	-0.0862803959954459	-1.24088351877606	1.06832272678517	0.291257174755333	10	exerciseintolerance
**Rudacks, 2022**	-0.302681237890228	-1.25253068045745	0.647168204676992	0.195064961391716	10	activity_complexI_III_IV

**Riley, 2010**	-0.379465955155562	-1.25583375479952	0.496901844488395	0.0689118681815837	10	activity_complexI_III_IV
**Sasarmanm, 2012**	-0.155107199206475	-1.12473404245619	0.814519644043244	0.224051843797636	10	activity_complexI_III_IV
**Riley, 2013**	-0.314370298614748	-1.30953180397271	0.680791206743218	0.202352636890343	10	activity_complexI_III_IV
**Shahni, 2013**	-0.155107199206475	-1.12473404245619	0.814519644043244	0.224051843797636	10	activity_complexI_III_IV
**Ardissone, 2014**	-0.236976217467799	-1.24435492098516	0.770402486049564	0.239946471969671	10	activity_complexI_III_IV
**Nakajima, 2014**	-0.155107199206475	-1.12473404245619	0.814519644043244	0.224051843797636	10	activity_complexI_III_IV
**Sommerville, 2017**	-0.378666354746655	-1.3819563100995	0.624623600606187	0.141177241115661	10	activity_complexI_III_IV
**Riley, 2018**	0.19793340233775	-0.526692529872712	0.922559334548212	0	10	activity_complexI_III_IV
**Smith, 2018**	-0.302681237890228	-1.25253068045745	0.647168204676993	0.195064961391716	10	activity_complexI_III_IV
**Carreño-Gago, 2021**	-0.113974954327576	-1.05801990569711	0.830069997041963	0.18641031566684	10	activity_complexI_III_IV
**Rudacks, 2022**	-1.3560679609928	-2.20080800436432	-0.51132791762128	0	10	inc_serum_ketones
**Riley, 2010**	-1.27887072349241	-2.11800236746275	-0.439739079522064	0	10	inc_serum_ketones
**Sasarmanm, 2012**	-1.3560679609928	-2.20080800436432	-0.51132791762128	0	10	inc_serum_ketones
**Riley, 2013**	-1.52606354156102	-2.36608065707011	-0.686046426051927	0	10	inc_serum_ketones
**Shahni, 2013**	-1.3560679609928	-2.20080800436432	-0.51132791762128	0	10	inc_serum_ketones
**Ardissone, 2014**	-1.31226558230685	-2.16003044944502	-0.464500715168679	0	10	inc_serum_ketones
**Nakajima, 2014**	-1.53303695661052	-2.18332408779474	-0.882749825426303	0	10	inc_serum_ketones
**Sommerville, 2017**	-1.2120214851312	-2.00848482864706	-0.415558141615343	0	10	inc_serum_ketones
**Riley, 2018**	-1.12107920403109	-1.79772233950461	-0.44443606855758	0	10	inc_serum_ketones
**Smith, 2018**	-1.3560679609928	-2.20080800436432	-0.51132791762128	0	10	inc_serum_ketones
**Carreño-Gago, 2021**	-1.31226558230685	-2.16003044944502	-0.464500715168679	0	10	inc_serum_ketones
**Rudacks, 2022**	-1.07197861558501	-1.89125044409526	-0.252706787074762	0	10	inc_alanine
**Riley, 2010**	-1.17015752937083	-2.00929911140932	-0.331015947332339	0	10	inc_alanine
**Sasarmanm, 2012**	-1.07197861558501	-1.89125044409526	-0.252706787074762	0	10	inc_alanine
**Riley, 2013**	-1.17015752937083	-2.00929911140932	-0.331015947332339	0	10	inc_alanine

**Shahni, 2013**	-1.20878939673957	-1.91148816066625	-0.50609063281289	0	10	inc_alanine
**Ardissone, 2014**	-1.03620609431192	-1.8509070948445	-0.221505093779345	0	10	inc_alanine
**Nakajima, 2014**	-1.20878939673957	-1.91148816066625	-0.50609063281289	0	10	inc_alanine
**Sommerville, 2017**	-0.956836363917543	-1.7148741186197	-0.19879860921538	0	10	inc_alanine
**Riley, 2018**	-0.77300707935749	-1.51967484369943	-0.0263393150155524	0	10	inc_alanine
**Smith, 2018**	-1.07197861558501	-1.89125044409526	-0.252706787074762	0	10	inc_alanine
**Carreño-Gago, 2021**	-1.03620609431192	-1.8509070948445	-0.221505093779345	0	10	inc_alanine
**Rudacks, 2022**	1.60883087840302	0.776786040605356	2.44087571620068	0	10	onset_infancy
**Riley, 2010**	1.52606354156102	0.686046426051927	2.36608065707011	0	10	onset_infancy
**Sasarmanm, 2012**	1.80709090185215	1.25281397129064	2.36136783241366	0	10	onset_infancy
**Riley, 2013**	1.52606354156102	0.686046426051927	2.36608065707011	0	10	onset_infancy
**Shahni, 2013**	1.60883087840302	0.776786040605356	2.44087571620068	0	10	onset_infancy
**Ardissone, 2014**	1.56226510361211	0.718720173731556	2.40581003349267	0	10	onset_infancy
**Nakajima, 2014**	1.60883087840302	0.776786040605356	2.44087571620068	0	10	onset_infancy
**Sommerville, 2017**	1.45262330738448	0.647682360449265	2.2575642543197	0	10	onset_infancy
**Riley, 2018**	1.35157922366497	0.660955082219362	2.04220336511057	0	10	onset_infancy
**Smith, 2018**	1.60883087840302	0.776786040605356	2.44087571620068	0	10	onset_infancy
**Carreño-Gago, 2021**	1.56226510361211	0.718720173731556	2.40581003349267	0	10	onset_infancy
**Rudacks, 2022**	1.30304233414303	0.949060002395062	1.65702466589101	0	10	anemia
**Riley, 2010**	1.25068719503901	0.917287222228621	1.5840871678494	0	10	anemia
**Sasarmanm, 2012**	1.30304233414303	0.949060002395062	1.65702466589101	0	10	anemia
**Riley, 2013**	1.25068719503901	0.917287222228621	1.5840871678494	0	10	anemia
**Shahni, 2013**	1.30304233414303	0.949060002395062	1.65702466589101	0	10	anemia
**Ardissone, 2014**	1.2732447791558	0.921654041467042	1.62483551684456	0	10	anemia
**Nakajima, 2014**	1.30304233414303	0.949060002395062	1.65702466589101	0	10	anemia
**Sommerville, 2017**	1.49236999993098	1.26600605541845	1.71873394444352	0	10	anemia
**Riley, 2018**	1.16529285562607	0.796447035858575	1.53413867539356	0	10	anemia
**Smith, 2018**	1.30304233414303	0.949060002395062	1.65702466589101	0	10	anemia

Carreño-Gago, 2021	1.2732447791558	0.921654041467042	1.62483551684456	0	10	anemia
Rudacks, 2022	1.06771952644566	-0.152012769414723	2.28745182230604	0.174226178960906	10	anemia_onset_infancy
Riley, 2010	0.979132155344815	-0.227574278613449	2.18583858930308	0.146361638395206	10	anemia_onset_infancy
Sasarmanm, 2012	1.28430293879749	0.178397815535957	2.39020806205901	0	10	anemia_onset_infancy
Riley, 2013	0.979132155344815	-0.227574278613449	2.18583858930308	0.146361638395206	10	anemia_onset_infancy
Shahni, 2013	1.06771952644566	-0.152012769414724	2.28745182230604	0.174226178960906	10	anemia_onset_infancy
Ardissone, 2014	1.01579704739086	-0.201529786846667	2.23312388162838	0.164694399147277	10	anemia_onset_infancy
Nakajima, 2014	1.06771952644566	-0.152012769414724	2.28745182230604	0.174226178960906	10	anemia_onset_infancy
Sommerville, 2017	0.91015398465938	-0.256288749114787	2.07659671843355	0.0789666355493703	10	anemia_onset_infancy
Riley, 2018	0.816657964675326	-0.249380849648149	1.8826967789988	0	10	anemia_onset_infancy
Smith, 2018	1.28430293879749	0.178397815535956	2.39020806205901	0	10	anemia_onset_infancy
Carreño-Gago, 2021	1.36199723849032	0.339961219136031	2.38403325784461	0	10	anemia_onset_infancy
Rudacks, 2022	0.580832152973487	0.0706165275832644	1.09104777836371	0	10	transfusion_dependent
Riley, 2010	0.534872006253027	0.0715856973156619	0.998158315190393	0	10	transfusion_dependent
Sasarmanm, 2012	0.674123127749186	0.233951394246728	1.11429486125164	0	10	transfusion_dependent
Riley, 2013	0.534872006253027	0.0715856973156619	0.998158315190393	0	10	transfusion_dependent
Shahni, 2013	0.580832152973487	0.0706165275832644	1.09104777836371	0	10	transfusion_dependent
Ardissone, 2014	0.5541648624714	0.0651482281810268	1.04318149676177	0	10	transfusion_dependent
Nakajima, 2014	0.580832152973487	0.0706165275832644	1.09104777836371	0	10	transfusion_dependent
Sommerville, 2017	0.604909870962232	0.0483936643619834	1.16142607756248	0	10	transfusion_dependent
Riley, 2018	0.824406861921727	0.227913636948948	1.42090008689451	0	10	transfusion_dependent
Smith, 2018	0.580832152973487	0.0706165275832645	1.09104777836371	0	10	transfusion_dependent
Carreño-Gago, 2021	0.65530076208344	0.146913581715175	1.1636879424517	0	10	transfusion_dependent
Rudacks, 2022	1.14245449344734	0.611759781168408	1.67314920572627	0	10	sideroblastic_phenotype
Riley, 2010	1.08973291597333	0.580195787577605	1.59927004436905	0	10	sideroblastic_phenotype

**Sasarmanm, 2012**	1.14245449344734	0.611759781168408	1.67314920572627	0	10	sideroblastic_phenotype
**Riley, 2013**	1.08973291597333	0.580195787577605	1.59927004436905	0	10	sideroblastic_phenotype
**Shahni, 2013**	1.14245449344734	0.611759781168409	1.67314920572627	0	10	sideroblastic_phenotype
**Ardissone, 2014**	1.11230917182305	0.587452575744284	1.63716576790182	0	10	sideroblastic_phenotype
**Nakajima, 2014**	1.14245449344734	0.611759781168409	1.67314920572627	0	10	sideroblastic_phenotype
**Sommerville, 2017**	1.49236999993098	1.26600605541845	1.71873394444352	0	10	sideroblastic_phenotype
**Riley, 2018**	0.956956440382098	0.405662776192232	1.50825010457196	0	10	sideroblastic_phenotype
**Smith, 2018**	1.14245449344734	0.611759781168409	1.67314920572627	0	10	sideroblastic_phenotype
**Carreño-Gago, 2021**	1.11230917182305	0.587452575744284	1.63716576790182	0	10	sideroblastic_phenotype
**Rudacks, 2022**	-1.13317773748214	-1.74923903703733	-0.517116437926941	0	10	neutropenia
**Riley, 2010**	-1.07756518423871	-1.67472130882689	-0.480409059650518	0	10	neutropenia
**Sasarmanm, 2012**	-1.13317773748214	-1.74923903703733	-0.517116437926941	0	10	neutropenia
**Riley, 2013**	-1.07756518423871	-1.67472130882689	-0.480409059650518	0	10	neutropenia
**Shahni, 2013**	-1.25635370150793	-1.71697865036481	-0.795728752651039	0	10	neutropenia
**Ardissone, 2014**	-1.26572182840169	-1.81835146175655	-0.71309219504684	0	10	neutropenia
**Nakajima, 2014**	-1.13317773748214	-1.74923903703733	-0.517116437926941	0	10	neutropenia
**Sommerville, 2017**	-1.03049392648495	-1.57520795529727	-0.485779897672636	0	10	neutropenia
**Riley, 2018**	-1.09567013345352	-1.85570808344258	-0.335632183464463	0	10	neutropenia
**Smith, 2018**	-1.13317773748214	-1.74923903703733	-0.517116437926941	0	10	neutropenia
**Carreño-Gago, 2021**	-1.10137630882712	-1.71235086107272	-0.490401756581527	0	10	neutropenia
**Rudacks, 2022**	-1.25635370150793	-1.71697865036481	-0.795728752651039	0	10	thrombocytopenia
**Riley, 2010**	-1.20190287252774	-1.6439911974966	-0.759814547558874	0	10	thrombocytopenia
**Sasarmanm, 2012**	-1.25635370150793	-1.71697865036481	-0.795728752651039	0	10	thrombocytopenia
**Riley, 2013**	-1.20190287252774	-1.6439911974966	-0.759814547558874	0	10	thrombocytopenia

**Shahni, 2013**	-1.25635370150793	-1.71697865036481	-0.795728752651039	0	10	thrombocytopenia
**Ardissone, 2014**	-1.39621310703309	-1.72468343008801	-1.06774278397816	0	10	thrombocytopenia
**Nakajima, 2014**	-1.25635370150793	-1.71697865036481	-0.795728752651039	0	10	thrombocytopenia
**Sommerville, 2017**	-1.15533623201542	-1.53157133249184	-0.779101131538999	0	10	thrombocytopenia
**Riley, 2018**	-1.28374778069833	-1.85225697924336	-0.715238582153309	0	10	thrombocytopenia
**Smith, 2018**	-1.25635370150793	-1.71697865036481	-0.795728752651039	0	10	thrombocytopenia
**Carreño-Gago, 2021**	-1.22532432850135	-1.68283151607859	-0.767817140924116	0	10	thrombocytopenia
**Rudacks, 2022**	-1.53303695661052	-2.18332408779474	-0.882749825426302	0	10	scoliosis
**Riley, 2010**	-1.45824761654953	-2.1101490957232	-0.806346137375862	0	10	scoliosis
**Sasarmanm, 2012**	-1.53303695661052	-2.18332408779474	-0.882749825426302	0	10	scoliosis
**Riley, 2013**	-1.72735065566918	-2.30464844958442	-1.15005286175394	0	10	scoliosis
**Shahni, 2013**	-1.53303695661052	-2.18332408779474	-0.882749825426303	0	10	scoliosis
**Ardissone, 2014**	-1.49083259517086	-2.14969542862659	-0.831969761715138	0	10	scoliosis
**Nakajima, 2014**	-1.53303695661052	-2.18332408779474	-0.882749825426303	0	10	scoliosis
**Sommerville, 2017**	-1.39245051064239	-1.99838644847519	-0.786514572809584	0	10	scoliosis
**Riley, 2018**	-1.30226395320934	-1.75854382353791	-0.845984082880773	0	10	scoliosis
**Smith, 2018**	-1.53303695661052	-2.18332408779474	-0.882749825426302	0	10	scoliosis
**Carreño-Gago, 2021**	-1.49083259517086	-2.14969542862659	-0.831969761715138	0	10	scoliosis
